# Functional genomic characterization of metallothioneins in brown trout (*Salmo trutta* L.). using synthetic genetic analysis

**DOI:** 10.1038/s41598-019-48303-0

**Published:** 2019-08-14

**Authors:** Josephine R. Paris, Jane Usher

**Affiliations:** 1 School of Biosciences, College of Life and Environmental Sciences, University of Exeter, Exeter, UK; 2 School of Life Sciences, University of Sussex, Brighton, UK

**Keywords:** Ecology, Genetic interaction

## Abstract

Metal pollution has made a significant impact on the earth’s ecosystems and tolerance to metals in a wide variety of species has evolved. Metallothioneins, a group of cysteine-rich metal-ion binding proteins, are known to be a key physiological mechanism in regulating protection against metal toxicity. Many rivers across the southwest of England are detrimentally affected by metal pollution, but brown trout (*Salmo trutta* L.) populations are known to reside within them. In this body of work, two isoforms of metallothionein (*MetA* and *MetB*) isolated from trout occupying a polluted and a control river are examined. Using synthetic genetic array (SGA) analyses in the model yeast, *Saccharomyces cerevisiae*, functional genomics is used to explore the role of metallothionein isoforms in driving metal tolerance. By harnessing this experimental system, *S. cerevisiae* is used to (i) determine the genetic interaction maps of *MetA* and *MetB* isoforms; (ii) identify differences between the genetic interactions in both isoforms and (iii) demonstrate that pre-exposure to metals in metal-tolerant trout influences these interactions. By using a functional genomics approach leveraged from the model yeast *Saccharomyces cerevisiae*, we demonstrate how such approaches could be used in understanding the ecology and evolution of a non-model species.

## Introduction

Metal pollution has made a significant impact on the earth’s ecosystems and yet tolerance to metals has evolved across the tree of life^1–4^. The southwest of England was once an important region for metal mining^5,6^. Although mining in the region has ceased, many rivers remain polluted with metals^7,8^. Despite evidence that these rivers contain metal concentrations known to affect fish physiology^9–11^; populations of resident brown trout (*Salmo trutta* L.) are found within them. Study of these metal-tolerant trout populations has shown that their genetic structure is different compared to fish from neighbouring control rivers, and that these demographic changes have occurred in association with periods of increased mining activity^12^. It has also been shown that metal-contaminated river water disrupts osmoregulation in metal-naïve trout^13,14^. Importantly, one of the key genetic mechanisms involved in metal tolerance includes the induction of metallothioneins^14,15^.

Metallothioneins (MTs) have been shown to play an important role in metal ion homeostasis and the detoxification of metals in fish^16–18^. MTs are a family of low molecular weight, cysteine-rich proteins, which consist of an especially high number of thiol groups and a lack of generic secondary structure motifs. MTs have the capacity to bind both physiological (e.g. copper, zinc) and xenobiotic (e.g. arsenic, cadmium) metals through the thiol group of cysteine residues, which can represent up to 30% of its constituent amino acids. Following the whole genome duplication (WGD) event in salmonids, two metallothionein isoforms exist in brown trout^19^. The brown trout does not yet have an assembled genome, yet in the closely related Atlantic salmon (*Salmo salar*), these two MT isoforms (hereafter *MetA* and *MetB*) are known to occur on different chromosomes (*MetA:* chromosome ssa16; *MetB:* chromosome ssa10) and the two isoforms have been shown to be differentially involved in response to metals^14,15^.

Synthetic Genetic Array (SGA) analysis is an unbiased method for the identification of synthetic genetic interactions, used for describing the interaction partners of proteins and specifically, in targeting synthetic genetic interactors. To date, SGA analysis has not been adopted as a general conceptual framework in functional genomics, largely because robust methods for identifying such interactions did not exist. However, in 2005, synthetic lethal interactions between mutations in the breast cancer susceptibility genes (BRCA1 or BRCA2) and members of the poly ADP ribose polymerase (PARP) enzyme superfamily were identified^20,21^. These studies demonstrate the validity of using synthetic lethality as a novel approach in identifying components of a genetic network. The utilization of SGA screens, using yeast as a bait to express an exogenous gene, highlights the robustness of this technique, and suggest its usage can be extended to other systems^22^. Although these experiments are performed in *S. cerevisiae*, SGA relies on the mating ability of this versatile yeast and such methods are therefore applicable to the analysis of interactions for metal tolerance in brown trout. Moreover, *S. cerevisiae* is known to be intrinsically sensitive to metal stress. This makes it an ideal model organism to test the effects of over-expressing genes that may be involved in metal tolerance.

Here, we aimed to explore whether a model organism, *S. cerevisiae*, could be used to explore the role of metallothioneins in conferring metal tolerance in a non-model species, the brown trout. Synthetic genetic array (SGA) analysis is used to examine two metallothionein isoforms isolated from brown trout occupying a metal-impacted river and a control river. In particular, aims were to (i) determine the genetic interaction maps of *MetA* and *MetB*; (ii) identify the subtle differences between these genetic interactions in both isoforms of the gene and (iii) establish whether pre-exposure to metal contaminants in metal-tolerant trout influences these interactions. We demonstrate how SGA analyses in *S. cerevisiae* can be adapted as a tool to investigate biological systems where functional genomics and high-throughput screening technologies are limited.

## Results and Discussion

### The expression of *MetA* and *MetB* in *Saccharomyces cerevisiae* confers metal tolerance

**Figure 1:**
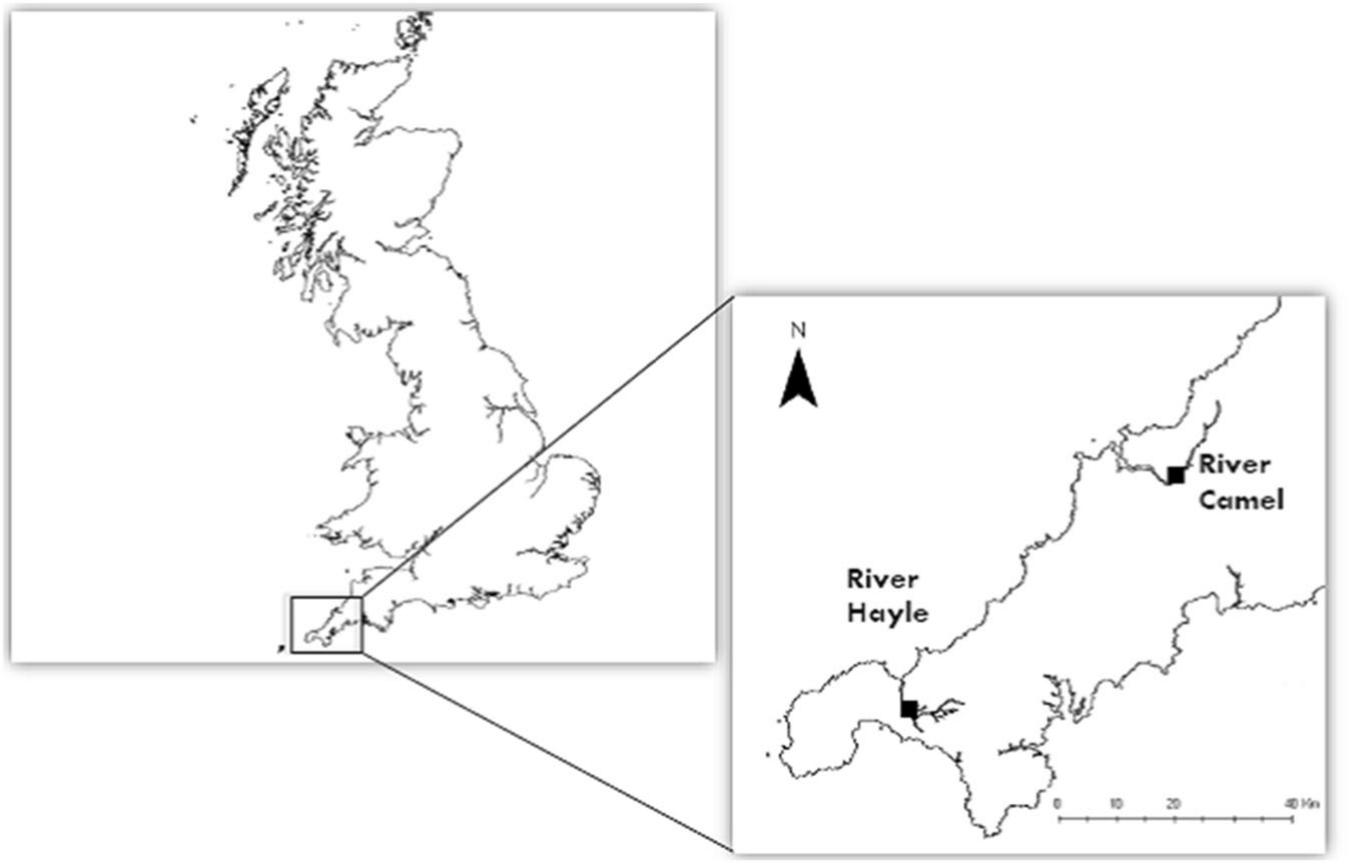
Map of River locations in Britain. Map of Britain, highlighting the rivers Hayle and Camel in southwest England. The River Hayle was used in this study as a region that has suffered from historical metal pollution. The River Camel was used as a relatively clean source in comparison. DNA from trout from both rivers was used to amplify the *MetA* and *MetB* genes.

**Figure 2:**
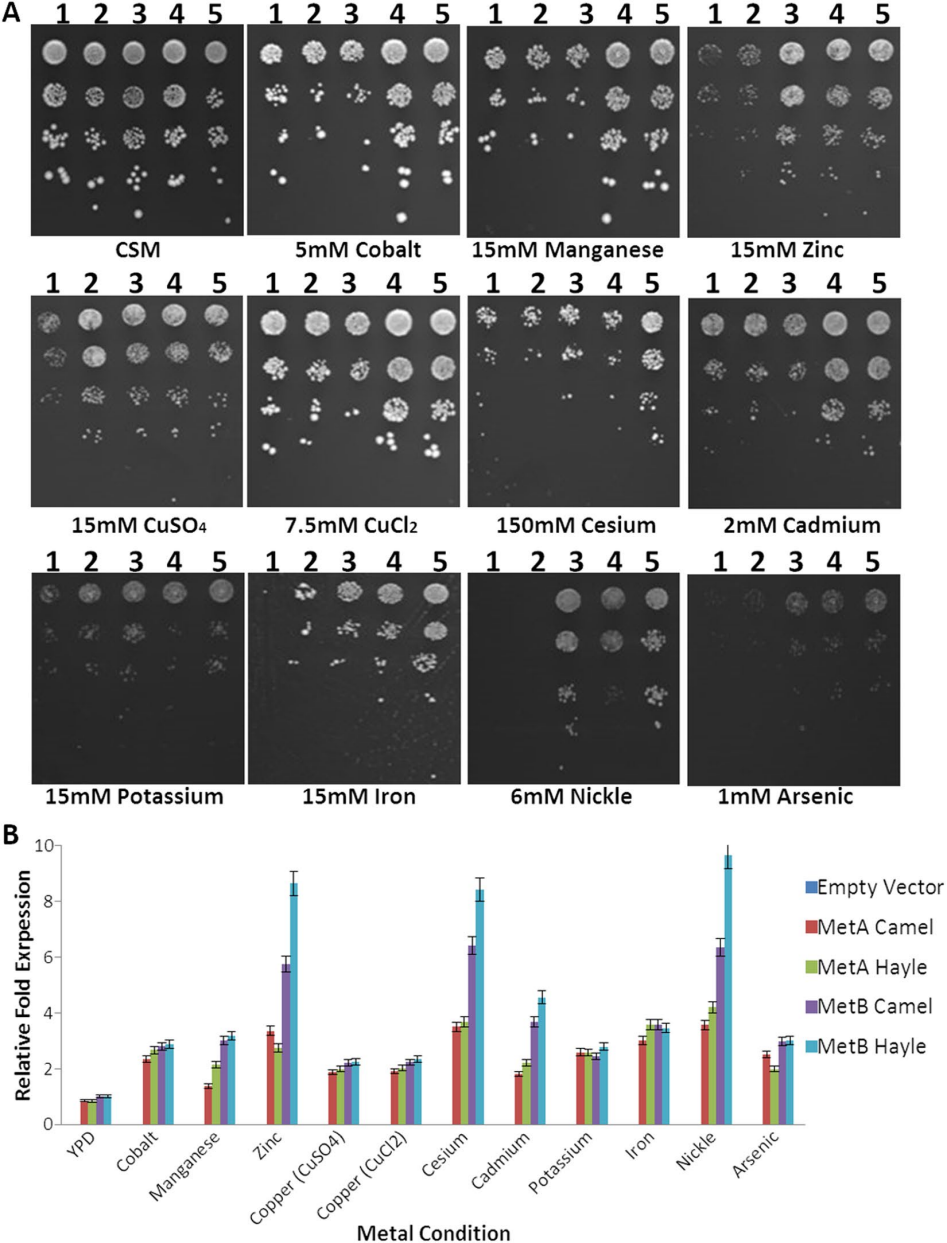
(**A**) Metal tolerant phenotypic screen. *S. cerevisiae* expressing one of the four genes *MetA_Cam*, *MetB_ Cam*, *MetA_Hay* and *MetB_Hay*) or an empty plasmid were tenfold serially diluted onto agar plates containing sub-lethal and lethal levels of metals (Table 1). The plates were incubated for 2 days at 30 °C. All of the MT genes amplified from the fish resulted in some level of metal tolerance; however, the most dramatic effects can be observed through the expression of *MetB* isolated from trout in the River Hayle. 1: *S. cerevisiae* transformed with empty plasmid p426_ccBd; 2: *S. cerevisiae* transformed with a plasmid containing *MetA* isolated from River Camel trout; 3: *S. cerevisiae* transformed with a plasmid containing the *MetA* gene from River Hayle trout; 4: *S. cerevisiae* transformed with a plasmid containing the *MetB* gene from River Camel trout; 5: *S. cerevisiae* transformed with a plasmid containing the MetB gene from River Hayle trout. (**B**) Levels of expression of MetA and MetB from the rivers Camel and Hayle when expressed in *S. cerevisiae*.

Genomic DNA isolated from brown trout from both the Hayle and Camel (Fig. 1A) was used as a template to amplify the *MetA* and *MetB* genes. Once the genes were transformed into *S. cerevisiae*, we performed real-time PCR to determine that the genes were being expressed (Fig. 2B) in tandem to a metal tolerant phenotypic screen (see Table 1 for metal concentrations used). The expression of *MetA* from both the Camel and Hayle fish resulted in a general increase in tolerance to metals when screened at lower concentrations (Fig. 2). Only the *MetB* from the Hayle fish offered an increased tolerance to zinc, cesium and nickel metals, which is also reflected by an increased expression of the gene under these conditions (Fig. 2A,B).

**Table 1:** Metal concentrations used in phenotypic screening of *S. cerevisiae* strains expressing *MetA* and *MetB*.

Metal	Concentrations screened (mM)
Cobalt^30^	5
Manganese^31^	15
Zinc^31–33^	15
Copper (CuSO4)^32–34^	15
Copper (CuCl2)^32–34^	7.5
Cesium (CsCl)^32,33,35^	150
Cadmium (CdCl)^32,33,36^	2
Potassium (KCl)^32^	15
Iron^32,33,37^	15
Nickle^32,33,38^	6
Arsenic^32,33,39^	1

### Genome-wide synthetic interaction analysis identifies novel regulators of metal tolerance in brown trout

The aim of utilizing SGA methodology was to systematically screen the yeast deletion mutant array to identify genetic interactions of metal tolerance conserved between *MetA* and *MetB* from two trout individuals (metal-tolerant Hayle and control Camel). The first goal was to construct the SGA starter strains with improved tolerance to the different metals (Fig. 2) Four plasmids were constructed, one expressing *MetA* from fish originating from the Camel river (*MetA_Cam*), one expressing *MetB* from the Camel trout (*MetB_Cam*) and two further plasmids containing *MetA* and *MetB* isolated from the metal-tolerant Hayle fish (*MetA_Hay* and *MetB_Hay*). As above, the *S. cerevisiae* strains containing the Met genes showed an improved growth phenotype on minimal media containing both sub-lethal and lethal concentrations of metals. The initial metal phenotypic screens (Fig. 2), demonstrated that the introduction of *MetA* and *MetB* from either the Camel or the Hayle fish improved the ability of the SGA query strain to grow on media containing lethal doses of metal.

Genome-wide SL-SGA screens with all four newly constructed query strains were performed. In brief, query strains were mated to the yeast deletion mutant array and the SGA methodology was used to incorporate the plasmids into the deletion mutants. Deletion mutants-plasmids combinations that resulted in synthetic lethal (SL), synthetic sick (SS) or improved growth were identified. The genetic interactions were confirmed, regardless of which screen the mutants were identified in. Each mutant was independently transformed with the four plasmids and a control empty plasmid, dot assays performed with the resulting growth scored (see Supplemental Table 2 for full list of interactions).

**Figure 3:**
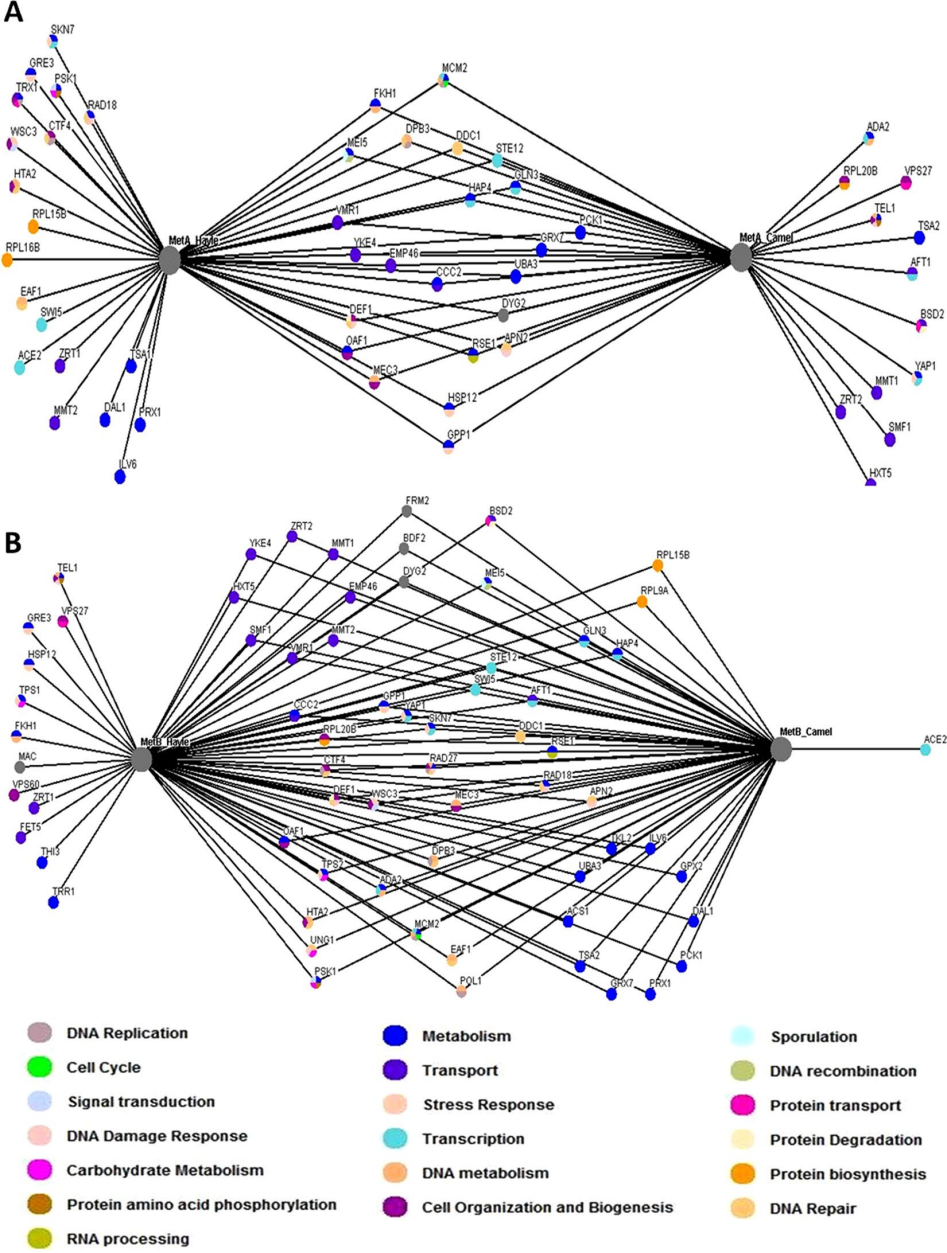
Genetic interaction network of *MetA* and *MetB* from Camel and Hayle trout. Genome-wide synthetic interaction SGA screens were performed using query strains that were transformed with (**A**) *MetA_Cam*, *MetA_Hay* and (**B**) *MetB_Cam*, *MetB_Hay*. Genes are represented by nodes that are colour-coded according to their *Saccharomyces* Genome Database (SGD) cellular roles (www.yeastgenome.org). Interactions are represented by edges. Deletion mutants that display a synthetic lethal (SL) interaction are detailed in Supplemental Table 2.

For the analysis of each of the SL-SGA screens performed, data for similar *MetA* and *MetB* interactors from both the Camel and the Hayle fish were paired. The SL-SGA screen for *MetA* from the Camel and Hayle resolved 38 and 43 genetic interactions respectively, of which 25 were common interactors (Fig. 3 and Supplemental Fig. 2 for interaction map of Hayle fish only). Of the 25 common interactors, all were synthetic sick (SS) interactions, showing a distinct inhibition of growth in combination with expression of *MetA* from either the Camel or Hayle fish.

For the *MetA_Cam*, a total of 12 unique genetic interactions were identified; 6 of which were phenotyped as synthetic lethal (SL) and 6 as synthetic sick (SS). Synthetic lethal interactions included: *ADA2*, *TSA2*, and *YAP1* with gene ontology (GO) terms including metabolism, transcription and DNA repair; and *MMT1*, *ZRT2* and *HXT5*, which are all involved in metal transport. *MMT1* and ZRT2 are metal transports for iron and zinc, respectively, whilst HXT5 has been shown to catalyse arsenic uptake^23^. In comparison, with *MetA_Hayle*, 19 unique interactions were observed; 7 of these as synthetic lethal (SL) and 12 as synthetic sick (SS). Synthetic lethal genetic interactions included: SKN7, GRE3, HTA2 with mixed GO terms encompassing metabolism, transcription, DNA repair and cell organisation and biogenesis; EAF1 which has been shown to be sensitive to cadmium^24^, SWI5, a paralog of ACE2, uniquely interacted with *MetB_Camel*, which is a transcription factor involved in activation of yeast metallothionein expression^25^, ZRT1, a high-affinity zinc transporter and TSA1 involved in the GO term metabolism, a cytoplasmic antioxidant involved in oxidative stress.

Of more interest to this study, were the differences observed in the genetic interaction maps for *MetB* between the Camel and Hayle fish. SGA analysis revealed a total of 56 genetic interactions for the Camel and 69 for the Hayle. Interestingly, GO term enrichment showed groups of genes involved in both metabolism and transport. Of the 56 Camel genetic interactions, only one was unique to the Camel, which showed a synthetic sick (SS) phenotype: *Ace2*, a transcription factor for cytokinesis and a known activator of CUP1 expression, a metallothionein that binds copper and mediates resistance to high concentrations of copper and cadmium in *S. cerevisiae*. The remaining interactions were common interactors with those identified in the *MetB_Hayle* screen, for which 3 were synthetic lethal in both *MetB_Camel* and *MetB_Hayle*: TSA2; a stress inducible cytoplasmic thioredoxin peroxidase, EAF1; a component of a histone acetyltransferase complex, which is also known to act for initiation of pre-meiotic DNA replication, and GRX7; which plays a role in oxidative stress response. The differences observed in the genetic interactions between *MetB_Camel* and *MetB_Hayle* is likely due to an amino-acid function change in the *MetB* of the Hayle fish, although in *in vivo*, multiple additional mechanisms are likely influencing these differences; for example, adaptive mutation in promoter regions, Cis-regulatory modules and copy number variation (CNVs). The SGA screen employed here is a first step in quantifying these differences and therefore provides future avenues to explore these mechanisms further.

In comparison, the *MetB_Hayle* SGA screen identified 60 genetic interactors, of which 12 were unique to the Hayle. Of these 12 interactions, 5 were SL and 7 SS were interactions, including 2 genes involved in metabolism (THI3 and TRR1); one gene involved with transport (FET5); one gene involved in protein transport (VSP60) and MAC1, which had an unknown GO term. In particular, the SL interactions (*TRR1; THI3; FET5; VPS60* and *MAC1)* unique to *MetB_*Hayle observed here, suggest that exposure to metals in these fish may drive complex genetic interactions for metal tolerance *in vivo*. Of particular interest is the SL interaction between *MetB_Hayle* and *MAC1*; a copper sensing transcription factor that is involved in the regulations of genes required for metal transport, in addition to a role in hydrogen peroxide induced transcription of *CTT1*, which is important in protection from oxidative damage^26^.

Both MT gene interaction maps were enriched for genes involved in DNA binding (average p-value 1.942e-11). The interactions from the Camel fish also showed further enrichment for genes involved in sequence-specific DNA binding transcription factor activity (p-value 5.202e-050) and metal ion binding (p-value 0.0008715), with the *MetB* dataset being enriched for peroxidase activity (p-value 7.271e-05). The interactions from the Hayle fish showed enrichment for genes involved in metal ion binding (p-value 7.758e-05), metal ion transmembrane activity (p-value 0.0001717) and peroxidase activity (p-value 0.0001294), which are all known to be important gene families involved in the detoxification of cells after exposure to high levels of toxic compounds, namely metal ions. The differences in the interaction maps suggest the potential different evolutionary pathways adopted by the two trout individuals. Previous research also points to differences in the roles that these two MT isoforms play in conferring metal tolerance in trout. In global transcriptome profiling of Hayle trout, *MetB* was among the most strongly up-regulated genes across four different tissues examined, whereas the *MetA* isoform was expressed at very low levels^15^. In cultured gill tissue exposed to metal-contaminated water from the Hayle, qPCR expression of both isoforms was elevated compared to controls, but different expression profiles were observed depending on the metal concentrations; with *MetB* showing elevated expression when concentrations were particularly high^14^. This suggests that the expression of the metallothionein isoforms contribute to metal tolerance in these trout, although other mechanisms are also likely operating.

## Conclusion

Through synthetic genetic array (SGA) screens, we have demonstrated how utilization of functional genomic technologies can be used to explore the evolution of metal tolerance in brown trout. Genetic interaction profiles were determined for two salmonid isoforms of metallothioneins, *MetA* and *MetB*, highlighting key differences in the genetic interactions of MT isoforms between metal-tolerant trout (Hayle) and fish from a control river (Camel). The expression of *MetA* from either the Hayle or Camel trout showed an increased tolerance to many of the metals screened for both, singly, or in combination. However, the most poignant finding from this first published screen of brown trout MT isoforms in an SGA analysis, are the differences in the genetic interactions between the *MetB* isoforms isolated from metal-tolerant trout, compared to control fish. This suggests that *MetB* is an important component of metal tolerance in these fish. In conclusion, the expertise of SGA has been used to identify both the conserved and unique synthetic genetic interactions across two MT isoforms in a non-model species. This provides an inventory of conserved MT synthetic lethal interactions that can be further investigated for their utility in contributing to the evolution of metal tolerance in brown trout.

## Methods

### DNA extraction and gene amplification

For the amplification and expression of the two isoforms of interest (*MetA* and *MetB*) in *S. cerevisiae*, DNA was isolated from two brown trout, one metal-tolerant fish from the River Hayle (50.14128, −5.411024) and a fish from a comparatively clean control river, the River Camel (50.579, −4.733) (Fig. 1). Fish were caught by electrofishing under Environment Agency authorisation (FR2 licencing), with assistance from the Westcountry Rivers Trust. Fish were anesthetised using benzocaine (10 g/100 mL ethanol) diluted 1:2000 in river water prior to fin-clip removal. Fin-clips were stored in 95% ethanol at 4 °C prior to DNA extraction. DNA was extracted from fin-clips using the QIAGEN DNeasy Blood and Tissue kit, following manufacturer guidelines.

Primers for amplification (Supplemental Table 3) of *MetA* and *MetB* were designed using the Atlantic salmon (*Salmo salar)* metallothionein sequences, obtained from NCBI (*MetA* Gene ID: 100136589 and *MetB* Gene ID: 100136581). PCR was carried out in 10 µl reactions consisting of 1 µl HF buffer; 0.2 µl gDNA; 0.2 µl 10 mM dNTPs; 0.3 µl DMSO; 1 µl F & R primer at 5 µM stock; 0.2 µl Phusion and 6.2 µl ddH2O. PCR cycling conditions were as follows: 98 °C for 30 s; 20 cycles of: 98 °C for 10secs; 70–50 °C for 30secs (−1 °C per cycle); 72 °C for 4 mins; 15 cycles of 98 °C for 10secs; 55 °C for30secs; 72 °C for 4 mins; with a final annealing at 72 °C for 10 mins with a hold at 4 °C. Product sizes were verified on 1.2% TAE gels prior to sequencing. Resultant PCR products were sequenced at Eurofins.

### Cloning of *MetA* and *MetB* into *S. cerevisiae*

For the genome-wide SGA screens and the metal tolerance phenotypic screening, the MATα query strain Y7092^27^ was transformed with one of the constructed plasmids; pDEST426-GPD::MetACam, pDEST426-GPD::MetAHay, pDEST426-GPD::MetBCam or pDEST426-GPD::MetBHay^28^, with *Cam* denoting the River Camel and *Hay* the River Hayle, briefly described herein. The gens were individually cloned into the pDONR221 entry vector using GATEWAY cloning technology^29^ and shuttled into a destination vector pAG426GPD-ccdB (AddGene) using LR clonase. Destination vectors carrying the MetA and MetB genes were transformed into *S. cerevisiae*. The empty vector pAG426GPD-ccdB was also transformed into *S. cerevisiae* as a control. Three independent transformants of each strain were collected.

### Confirming expression of MetA and MetB in *S. cerevisiae*

10 ml aliquots of cells at an OD of 0.6 at 600 nm were pelleted and RNA extraction was performed using a yeast RNA extraction kit (Masterpure Yeast RNA purification kit, Epicentre) following the manufacturer’s instructions. RNA quality was checked by electrophoresis on a denaturing gel, (1.2% agarose, 1X HEPES, 6% Formaldehyde). The RNA concentration was measured using a NanoDrop spectrophometer. Using iScript Reverse Transcription Supermix for RT-PCR (Bio-Rad), cDNA synthesis was performed following the manufacturer’s instructions. Primers used for qPCR are listed in Supplementary Table X, reactions were performed in the presence of SYBR Green (Bio-Rad) on a Bio-Rad CFXConnect Real-time System. Histograms represent the data from three biological replicates with error bars from the standard deviation of three biological replicates.

### Growth conditions and phenotypic screening

Cells were grown in standard YEP or synthetic complete (SC) media supplemented with glucose to a final concentration of 2% and metal concentrations as shown in Table 1. To assess growth under different metal stress conditions, wild-type cells containing an empty plasmid and transformed cells were grown in SC-uracil. Cells were grown to mid-log phase before phenotypic screens were performed. Dot assays were performed by spotting 5 µl of 10-fold serial dilutions (OD600 = 0.1, 0.01, 0.001, 0.0001) onto specified media, and sealed plates were incubated at 30 °C for 24 hours. All dot assay experiments were repeated in triplicate using three different isolates of each strain.

### SGA screening methodology

The deletion mutant library was robotically arrayed using a Singer RoToR HDA (Singer instruments). For the *MetA* and *MetB* genome-wide synthetic-lethal-SGA screens (SL-SGA), the MATα query strains Y7092^27^ were transformed with either pAG426GPD-ccdB_MetA or pAG426GPD-ccdB_MetB for *MetA* and *MetB*, respectively. Resulting query strains were mated with the MATa deletion mutant array and SGA methodology was used, (see Supplemental Table 1 and Supplemental Fig. 1 for workflow). To identify deletion mutants that displayed growth defects or advantages to the expression of *MetA* or *MetB*, all genome-wide screens were performed in triplicate at 30 **°**C. Growth was visually scored for three main criteria, slow growth (SS), lethality (SL) or suppression (S) after one day on final DMS plates. For verification, any putative genetic interactions had to be identified in a minimum of two out of the three replicates in any of the screens.

### Data analysis

The SGA screen potential hits were confirmed by transformation and selection on *Ura* media. Confirmed hits were analysed using the FunSpec web tool, (http://funspec.med.utoronto.ca/), where an input list of genes gives an output summary of functional classes, cellular localizations and phenotypes, which are enriched in the list (Supplemental Table 2). P-values were calculated using the hypergeometric distribution and represent the probability that the intersection of a given list with any given functional category occurs by chance. The Bonferroni correction divided the p-value threshold which would be deemed significant for an individual test by the number of tests conducted and therefore accounts for significance due to multiple testings over the categories of the database. Following the Bonferroni correction, only categories are displayed which the chance probability of enrichment is lower that the p-value (0.01).

## Supplementary information

Supplementary figures and tables

Supplementary Dataset 1

## Data Availability

The authors state that all data necessary for confirming the conclusions presented in the article are represented fully within the article.
