# Publisher Correction: Functional genomic characterization of metallothioneins in brown trout (*Salmo trutta* L.). using synthetic genetic analysis

**DOI:** 10.1038/s41598-019-56349-3

**Published:** 2019-12-24

**Authors:** Josephine R. Paris, Jane Usher

**Affiliations:** 1 School of Biosciences, College of Life and Environmental Sciences, University of Exeter, Exeter, UK; 2 School of Life Sciences, University of Sussex, Brighton, UK

Correction to: *Scientific Reports* 10.1038/s41598-019-48303-0, published online 14 August 2019

The Acknowledgements section in this Article was omitted. The Acknowledgements section should read:

“We would like to thank the Environment Agency, Westcountry Rivers Trust and the University of Exeter for the funding and support of Dr Paris’ PhD project on trout metal tolerance. Particular thanks to members of the Environment Agency and Westcountry Rivers Trust for organising permits for electrofishing, fieldwork and collection of trout tissue used in the experiments. Thanks also to Dr Jamie R. Stevens and Dr Eduarda Santos for conception of the PhD project, and also to Dr R. Andrew King, who assisted with gene identification and molecular laboratory work.”

