# Supplementary material for: Functional genomic characterization of metallothioneins in brown trout (Salmo trutta L.). using synthetic genetic analysis
Source: Sci Rep. 2019 Aug 14;9:11827. doi: 10.1038/s41598-019-48303-0 (PMC6694099; doi:10.1038/s41598-019-48303-0)
Supplement: Supplementary file 1 — Supplementary figures and tables [file 41598_2019_48303_MOESM1_ESM.pdf]

Functional genomic characterization of metallothioneins in brown trout (*Salmo trutta* L.). using synthetic genetic analysis.

Josephine R Paris[1, 2] and Jane Usher[1]

[1] School of Biosciences, College of Life and Environmental Sciences, University of Exeter, Exeter, UK

[2] School of Life Sciences, University of Sussex, Brighton, UK

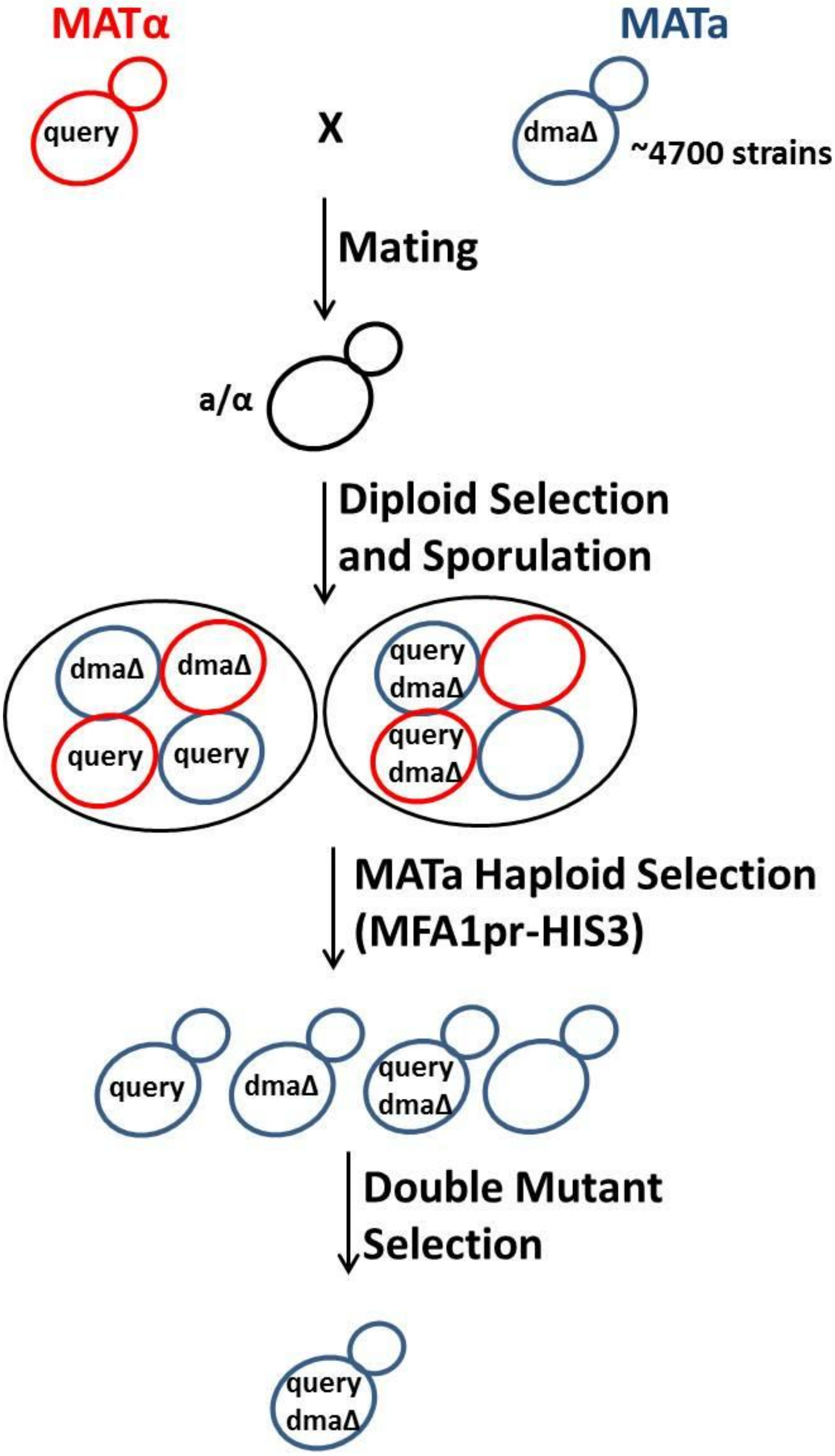

Supplemental Figure 1: Workflow for SGA screening.

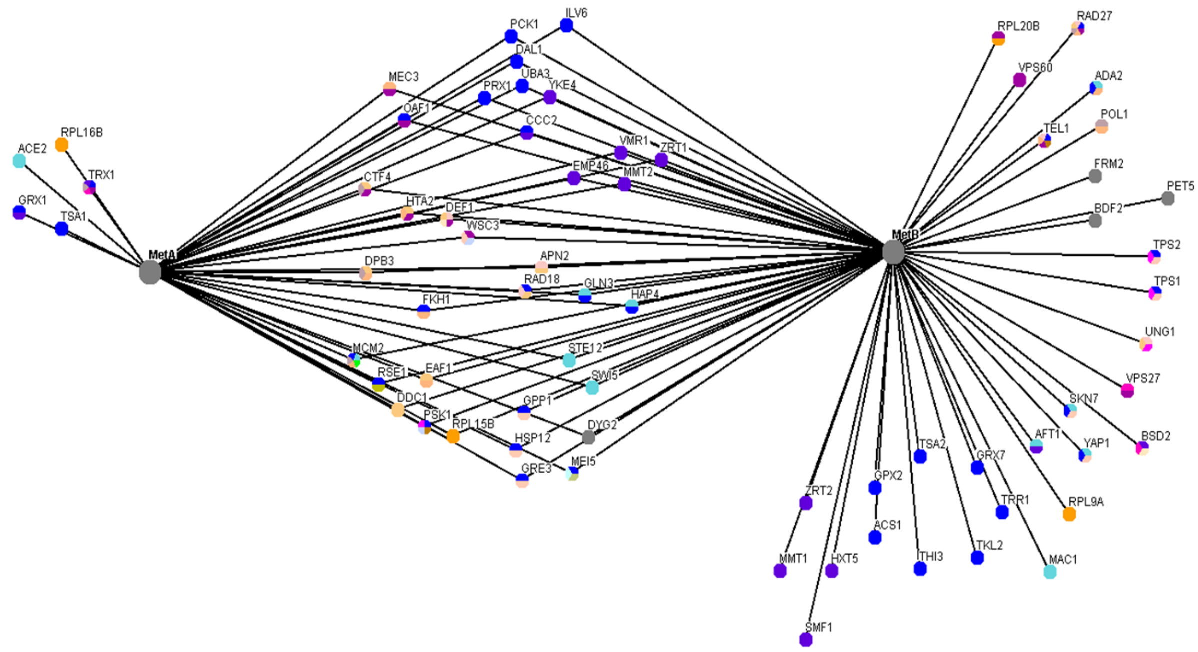

Supplemental Figure 2 Interaction map for MetA and MetB from the Hayle river only

Supplemental Table 1 Media and conditions used for SGA screen.

**URA3 system**							
**<u>Bait</u>**	**<u>S288c Deletion Array</u>**						
MATα	X MAT a						
		**Incubation**					
**Step**	**Media**	**Time**	**Temperature**				
Mating	YEPD + ADE	1 day	RT				
Diploid	SD-URA LYS	2 days	30°C				
SPO	SPO + Y.E. + glucose + a.a	5 days	22°C				
MATa haploid (1st)	SD-HIS ARG+CAN	2 days	30°C	MATa	can1Δ::MFA1pr-HIS3		
MATa haploid (2nd)	SD-HIS ARG+CAN	1 day	30°C	MATa	can1Δ::MFA1pr-HIS3		
kanR selection	SD(msg)-HIS ARG+CAN,G418	2 days	30°C	MATa	can1Δ::MFA1pr-HIS3	xxxΔ::kanR	
kanR URA selection	SD(msg)-HIS ARG URA+CAN,G418	1-2 days	30°C	MATa	can1Δ::MFA1pr-HIS3	xxxΔ::kanR	GOI::URA3

Supplemental Table 3: Primers used in this study

Primer	Sequence
MetA for	ACAAAAAAGCAGGCTTCCCTCACTGACAACAGCTGGTATCA
Met A rev	TACAAGAAAGCTGGGTCGATCCTTGTGAATGCTCCAAAAGTAAGTTA
Met B for	ACAAAAAAGCAGGCTTCCCTGACTGACAACAGCTGGTATCACAG
Met B rev	TACAAGAAAGCTGGGTCGATCCTTGTGAATGCTCTAAAAGTAAGTTT
Real-time PCR primers	
**Primer**	**Sequence**
NADH for	GTTTAGGGGCCAAATCCAAT
NADH rev	GCTTAGCACCCTGAGCAATC
MetA for	TCTTACAACTGGTGCATGCA
Met A rev	TGGATCCTTGTGAATGCTCCA
Met B for	AGGCTCTAGGATGACTTGGC
Met B rev	CTGCGTGTTGGCCATTAGAA
